# Nerve Injuries after Glenohumeral Dislocation, a Systematic Review of Incidence and Risk Factors

**DOI:** 10.3390/jcm12134546

**Published:** 2023-07-07

**Authors:** Alejandro Lorente, Gonzalo Mariscal, Carlos Barrios, Rafael Lorente

**Affiliations:** 1 Department of Traumatology and Orthopaedic Surgery, University Hospital Ramón y Cajal, 28034 Madrid, Spain; 2 Institute for Research on Musculoskeletal Disorders, School of Medicine, Valencia Catholic University, 46001 Valencia, Spain; 3 Department of Orthopedic Surgery and Traumatology, University Hospital of Badajoz, 06006 Badajoz, Spain

**Keywords:** nerve injury, glenohumeral dislocation, systematic review, incidence, functional outcomes, recovery

## Abstract

Glenohumeral dislocation is a common shoulder injury that can result in nerve injury. However, the full impact of these injuries on patient function and recovery remains unclear. This systematic review aimed to determine (1) the incidence, (2) risk factors, and (3) functional outcomes following nerve injuries after glenohumeral dislocation. The study followed PRISMA guidelines and used the PICO strategy. PubMed, EMBASE, Scopus, and Cochrane Collaboration Library databases were searched for studies. Two reviewers independently assessed the study eligibility, and data extraction was conducted by two authors. The quality of included studies was assessed using the Methodological Index for Non-Randomized Studies (MINORS) criteria. Thirteen studies comprising 17,087 patients were included. The incidence of nerve injury ranged from 0.4% to 65.5%, with the axillary nerve being most commonly affected. The time to reduction did not significantly affect the incidence of nerve injury. The mechanism of injury, the affected side, associated injuries, and recovery time were found to be potential risk factors for nerve injury. Motor recovery was incomplete in many patients, and sensory recovery was less complete. By synthesizing the available evidence, this systematic review underscores the importance of considering nerve injury in the management of patients with glenohumeral dislocations. Future research can build on these findings to develop targeted prevention and treatment approaches that optimize patient outcomes.

## 1. Introduction

Anterior shoulder dislocation is the most common dislocation [1], with a frequency of 1–2% in the general population [2,3,4]. Shoulder dislocations often occur during high-impact injuries, especially in athletes and those who participate in contact sports [3]. Forceful abduction and external rotation of the arm during these injuries can cause the humeral head to be forcibly dislocated from the glenoid fossa. These dislocations can cause damage to the soft tissues surrounding the joint, including the rotator cuff tendons, nerves in the area, labrum, and humeral or glenoid bone lesion [3,4]. However, there is limited evidence regarding this association following nerve injury [5,6,7]. The close relationship between the glenohumeral joint and the brachial plexus makes nerve injury a potential complication of shoulder dislocation, with the axillary nerve being the most frequently affected nerve [5,6]. The axillary nerve runs inferior to the shoulder joint, providing motor innervation to the deltoid and teres minor muscles as well as sensory innervation to the skin over the inferior portion of the deltoid [5,6]. Damage to this nerve may cause weakness in abduction and flexion of the arm as well as loss of sensation over the deltoid. The mechanism of nerve injury depends on the position of the upper extremity during the dislocation and reduction. Forced abduction and external rotation of the arm during dislocation or overly aggressive manipulation during reduction may stretch or compress the axillary nerve, leading to neurapraxia or more severe nerve injury [6]. A proper reduction technique is critical to avoid iatrogenic nerve damage. The diagnosis of nerve injury is usually clinical, based on a thorough neurological examination assessing strength, sensation, and deep tendon reflexes. Imaging techniques such as ultrasound, electromyography (EMG), and magnetic resonance imaging (MRI) may be useful if there are signs of severe or persistent nerve injury [8]. In particular, EMG remains controversial, with some studies showing a higher incidence of nerve lesions after shoulder dislocation [6,9]. Although most nerve injuries recover spontaneously over time, early diagnosis of significant nerve damage is critical to prevent chronic pain, shoulder instability, and impaired function [10,11]. Therefore, the objectives of this study were to review the available evidence regarding nerve injury associated with shoulder dislocation, assess the incidence and functional outcomes of these injuries, and identify predictive risk factors for use in prevention or early diagnosis.

## 2. Material and Methods

### 2.1. Eligibility Criteria

**Figure 1:**
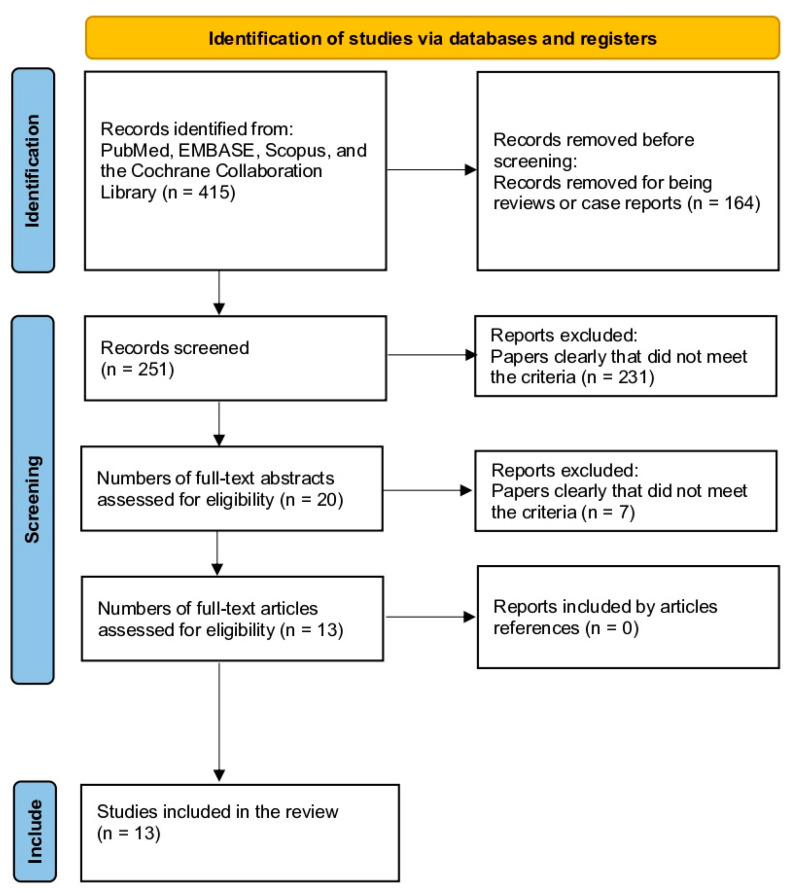
Study selection flow diagram (Preferred Reporting Items for Systematic Reviews and Meta-Analyses).

This study had a written protocol with review questions, search strategy, inclusion/exclusion criteria, and a risk of bias assessment. This study followed the Preferred Reporting Items for Systematic Reviews and Meta-Analyses (PRISMA) guidelines (Figure 1) [12]. The research question was formulated using the PICO strategy: (P) adult patients with primary or sequential anterior glenohumeral dislocation and age > 18 years; (I) patients with a diagnosis of nerve injury; (C) control patients without nerve injury for comparison; (O) outcomes included incidence of nerve injury in patients with shoulder dislocation, functional outcomes, risk factors, and associations. Nerve injury was diagnosed clinically or using electromyography (EMG) with the diagnostic method specified in Table 1. Papers relating to pediatric patients, epilepsy, posterior dislocation, or luxatio erecta, non-English language papers, case reports, previous reviews, duplicate data, incomplete data, or articles that did not share data were excluded (Table 1).

Missing data were considered when complete information was not available for all studies included in the analysis. This can happen when certain relevant variables are not reported in some studies or when data are incomplete or lost during the data collection process.

### 2.2. Information Sources

The search strategy employed relevant MeSH terms including ‘Glenohumeral Dislocation’, ‘Nerve Injuries’, ‘Incidence’, ‘Functional Outcome’, ‘Risk Factors’, and ‘Associations’ to conduct a comprehensive search for relevant articles. A systematic search of the literature using PubMed, EMBASE, Scopus, and the Cochrane Collaboration Library databases was carried out. Language was limited to English. There was no restriction to the year of publication. Studies of interest that appeared in the references of the included studies in the first search were also evaluated.

### 2.3. Search Methods for Identification of Studies

Two reviewers independently agreed on the selection of eligible studies and achieved consensus on which studies to include. An initial screening of titles and abstracts was performed to eliminate studies that were obviously outside the scope of the review. In cases of uncertainty based on title or abstract, the full text of each article was examined for further evaluation. If consensus was not reached, a third review author was asked to complete the data extraction form and discuss the article with the other two authors until consensus was reached. All disagreements were resolved by discussion. We consulted senior shoulder orthopedic surgeons of our orthopedic departments to assess which variables would be of most interest, as well as to evaluate the shortcomings of previous studies.

### 2.4. Data Extraction and Data Items

For data extraction, two authors independently reviewed the studies and extracted baseline characteristics, including region, study type, follow-up period, age, gender, diagnostic criteria, and inclusion criteria. The primary variables were the presence of nerve injury and the type of nerve affected, while secondary variables included characteristics associated with dislocation, such as recovery time, reduction time, type of deficit (motor or sensory), recurrence of dislocation, trauma energy, mechanism of action, affected side, and associated injuries. In addition, unplanned variables that were subsequently considered important by experts in the field were also included in the analyses.

### 2.5. Assessment of Risk of Bias in Included Studies

The quality of the included studies was assessed independently by two authors using the Methodological Index for Non-Randomized Studies (MINORS) criteria (Table 2) [21]. The maximum score is 24 for comparative studies and 16 for noncomparative studies. For noncomparative studies, scores of 0–4 correspond to very low quality, 5–7 to low quality, 8–12 to fair quality, and ≥13 to high quality. For comparative studies, scores of 0–6 correspond to very low quality, 7–10 to low quality, 11–15 to fair quality, and ≥16 to high quality.

### 2.6. Assessment of Results

Given the heterogeneity in methodology and reporting of results across the studies, a qualitative review was conducted.

## 3. Results

### 3.1. Study Selection

The initial search yielded 415 results, and, after excluding review studies and case reports, a total of 251 articles were reviewed. After screening titles and abstracts, 20 articles met the inclusion criteria. Upon full text review, seven studies were excluded, leaving a total of 13 studies for inclusion. No additional studies meeting the inclusion criteria were found upon reviewing the references of the included articles. In total, 13 studies were included in the systematic review [5,7,9,11,12,13,14,15,16,17,18,19,20] (Figure 1).

### 3.2. Study Characteristics

Table 1 presents the baseline characteristics of the included studies, which comprised a total of 13 articles with 17,087 patients. The mean age of patients with shoulder dislocation ranged from 35 to 64 years, and the studies included two cohort studies (one retrospective and one prospective), 10 clinical series (seven retrospective and three prospective), and one cross-sectional study. The percentage of women ranged from 26% to 73%.

### 3.3. Incidence of Nerve Injury

The incidence of nerve injury in the included studies ranged from 0.4% to 65.5%, with studies that included EMG reporting a higher incidence of nerve injury ranging from 39.8% to 65.5%. The mean age of patients with nerve injury ranged from 35 to 52 years. Table 3 shows the frequency of involvement of each individual nerve, with a total of 826 nerve lesions observed. The most common injury was to the axillary nerve (35.0%), followed by the radial nerve (12.8%), suprascapular nerve (11.1%), musculocutaneous nerve (10.3%), median nerve (10.1%), global plexus (9.4%), ulnar nerve (9.3%), supraclavicular (1.0%), infraclavicular (0.9%), and posterior and medial cord (0.1%).

### 3.4. Risk Factors

Hardie et al. reported that the time until reduction was 102 min (range 49–131 min), and no significant difference was found compared to a control group without nerve injury [5]. Gutkowska et al. showed that a time to reduction of less than six hours occurred in 34/73 (46.6%) patients with nerve injury, while one of more than seven hours occurred in 7/73 (9.6%) [14]. Additionally, the influence of a first dislocation was reported by Hardie et al., with the probability of nerve injury in the first episode being up to five times higher compared to in a control group (OR 5.94; 95% CI 1.50–23.46; *p* = 0.01) [5].

There was controversy regarding the energy of the mechanism, with Hardie et al. [5] and Kosiyatrajul et al. [7] reporting a higher proportion of patients with nerve injury in the high-energy group, while Gutkowska et al. [14] found significant differences in favor of the low-energy mechanism. Jordan et al. [15] also reported a higher number of patients with nerve injury in the low-energy group. There were no significant differences observed with respect to the affected side, with Gutkowska et al. [14] observing the right side being affected in 40 patients (55%) and the left side in 33 patients (45%), and Kosiyatrakul et al. [7] reporting similar proportions on both sides: right: 7, left: 6, and 1 bilateral.

### 3.5. Associated Injuries

Table 4 shows the most frequent associated injuries observed in the included studies. Regarding greater tuberosity fractures, the age of patients ranged from 32.8 years (25–38) (16) to 56.3 years (54.0 to 58.6) [11], and the male-to-female ratio was 0.9:1 [11]. However, for rotator cuff tears, the mean age was higher, 53.9 years (49–58) [13] and 63.0 years (59.8 to 66.3) [11], and the male-to-female ratio was 1.2:1 [11]. Furthermore, Gutkowska et al. [14] reported humeral fractures in 2/73 (2.7%) patients, and Travlos et al. [20] observed fractures in the humerus (2/28), clavicle (1/28), scapula (3/28), and combined clavicle and scapula (1/28) [14,20].

### 3.6. Mechanism of Injury

The most frequent mechanisms of shoulder dislocation associated with nerve injury were casual falls and traffic accidents. Jordan et al. reported motorbike accidents in 2/28 (7.1%) patients, a bicycle accident in 1/28 (3.6%), and falls from height in 25/28 (89.3%) (17). Travlos et al. observed minor falls in 9/28 (32.1%) patients (treated conservatively), major falls in 6/28 (21.4%), motor vehicle accidents in 4/28 (14.2%), direct blows to the shoulder in 3/28 (10.7%), and other mechanisms in 3/28 (10.7%) [20]. Kosiyatrakul et al. reported simple falls in 5/14 (35.7%) patients, car accidents in 3/14 (21.4%), motorcycle accidents in 3/14 (21.4%), ski accidents in 1/14 (7.1%), injuries obtained while lifting heavy objects in 1/14 (7.1%), and falls from height in 1/14 (7.1%) (7). Yeap et al. observed motor vehicle accidents in 2/11 (18.2%) patients, spontaneous dislocations in 2/11 (18.2%), and falls in 7/11 (63.6%) [9].

### 3.7. Functional Outcomes

Regarding motor recovery, Hardie et al. observed persistent motor injury in up to 8/14 (57.2%) patients at the end of follow up, with complete loss of function in 4/14 (28.6%) (three global plexus and one axillary nerve) and partial loss in 4/14 (28.6%) (three global plexus and one posterior and medial cord) [5]. Tiefenboeck et al. [18] observed that out of 35 patients with initial nerve injury, 25 were left with motor deficits at the end of follow up. The affected nerve was not reported [18]. Jordan et al. reported that at the end of follow up, 2/22 (9.1%) patients with axillary nerve injury, 6/17 (35.3%) with ulnar nerve injury, 1/15 (6.7%) with radial nerve injury, 5/14 (35.7%) with musculocutaneous nerve injury, and 1/12 (8.3%) with median nerve injury did not recover [15]. Kosiyatrakul et al. observed that recovery of the supraspinatus, deltoid, elbow flexors, elbow extensors, wrist extensors, and finger extensors was complete or nearly complete at the final follow up [7]. Yeap et al. assessed the recovery of nerve function after dislocation reduction and observed that only 3 out of 11 patients recovered, with one patient taking longer to recover full motor function [9].

Regarding sensory recovery, Hardie et al. observed a lack of complete sensory function recovery in 2/14 (14.3%) (one axillary nerve and one ulnar nerve) patients and a partial lack of recovery in 9/14 (64.3%) (six global plexus, one axillary nerve, one radial nerve, and one posterior and medial cord) [5]. Tiefenboeck et al. observed that out of 52 patients with sensory impairment at the end of follow up, 21 patients (40.4%) maintained a sensory deficit. The affected nerve was not reported [18].

## 4. Discussion

The incidence of nerve injury associated with shoulder dislocation varies considerably among studies [6,11,13,19]. Retrospective studies tend to report lower incidence rates than prospective studies, which may be attributed to differences in diagnostic methods. Studies utilizing electrodiagnostic testing, such as electromyography (EMG), often detect higher rates of nerve injury, likely because of the increased sensitivity and accuracy of these tests in assessing nerve conduction and muscle activity [6,19]. Additionally, the available evidence indicates that the time taken to reduce the dislocation is not a predictive factor for nerve injury outcomes. These findings suggest that nerve injury may be more related to the violence or energy of the dislocation, which is reinforced by observing how the first dislocation increased the probability of nerve injury by up to five times, as well as the high-energy mechanism observed in comparative studies with control groups without nerve injury [5]. This observation aligns with the idea that initial trauma might cause more severe damage to the surrounding tissue, including nerves, leading to a higher likelihood of injury. Regarding the traumatic mechanism, some studies suggest that the low-energy mechanisms produced in older patients may be associated with a higher risk of nerve injury [9,16]. This may be attributed to age-related physiological changes, such as a decline in muscle mass and strength, as well as decreased flexibility and elasticity of connective tissues [22], which could render older patients more susceptible to nerve injury even in low-energy disorders. It was also observed that primary dislocation with higher energy presents a higher risk of complications. This could be due to fractures or associated hematomas [23]. Fractures and hematomas may increase the likelihood of nerve injury because they can cause additional compression or tension on the nerves, leading to damage or dysfunction.

This review highlights the importance of evaluating concomitant injuries with shoulder dislocation as their presence may indicate an increased likelihood of nerve damage. Greater tuberosity fractures were the most common associated injuries, occurring in approximately 10% of patients with nerve injury, followed by rotator cuff tears. The correlation between fractures/soft tissue injuries and nerve damage may be explained by the higher energy of injury required to cause multiple injuries as well as decreased tissue compliance in older patients [24,25]. Comprehensive physical examination, imaging, and possibly electrodiagnostic studies are warranted following shoulder dislocation to assess nerve injury and associated conditions. Their detection is critical for prompt treatment and prevention of long-term disability. Non-operative management such as sling immobilization, physical therapy, and pain control may be indicated for minor nerve injuries, whereas more severe cases may require surgical exploration and nerve repair or grafting [26,27]. Close monitoring of nerve function is required, regardless of the initial management approach [28].

Nerve injuries can often go undetected following shoulder dislocation, which may have medicolegal implications if loss of function results. The axillary nerve is the most commonly affected nerve, consistent with other reports, while more global brachial plexus injuries tend to occur with higher-energy mechanisms. Most nerve injuries are neurapraxias or transient disruptions in nerve conduction, which typically resolve with conservative management. However, more severe injuries such as axonotmesis and neurotmesis may require surgical intervention to repair or graft the nerve to prevent permanent loss of function [29].

Functional outcomes following nerve injury can be significant, resulting in a large loss of function, reduced quality of life, and increased costs [24]. Complete or partial loss of function, as well as sensory loss, has been reported in some studies, reaching or being close to 50% in many cases [5,18]. It is unclear whether advanced age influences the results, as older patients may have lower daily life requirements. There is also controversy regarding the need for follow-up consultations for deficits seen in the emergency department, as some studies reported nerve injury as favorable [6,7], while others raised concerns about loss of function [18]. This inconsistency in the literature underscores the importance of individualized patient care and careful monitoring of recovery from nerve injury. Motor recovery tends to have a better prognosis than sensory recovery after nerve injury. However, the relationship between motor and sensory deficits is not always straightforward and may be due to differences in nerve physiology and injury severity. Visser et al. proposed obtaining electromyography within three weeks of injury for optimal diagnosis and to guide treatment [6]. Early electrodiagnostic testing can accurately detect nerve injuries, even in the absence of clinical deficits, and determine the appropriate management, rehabilitation needs, and prognosis. Nerve injury requires a coordinated multidisciplinary approach to optimize outcomes. Orthopedic surgeons, neurologists, physical/occupational therapists, and other specialists may be involved in the proper diagnosis and management of nerve damage.

This study had several limitations. First, the individual studies included in the review showed high heterogeneity, which made it difficult to compare them through a meta-analysis. Additionally, some of the studies were published before 2000, and the incidence of some injuries may have changed over time. Furthermore, the diagnostic criteria varied between clinical, electromyography (EMG), and magnetic resonance imaging (MRI) or were unspecified, which could potentially have biased the pooled estimates. Moreover, the design of the studies and the small sample size in some cases may have led to bias in terms of incidence inference. It was also challenging to establish which exact type of glenohumeral dislocation was included in the studies, as the information was sometimes given in words and was difficult to accurately evaluate.

## 5. Conclusions

In conclusion, the incidence of nerve injury after shoulder dislocation varies widely, ranging from 0.4% to 65.5%. The most common nerve injuries that should be considered during patient examinations were identified. Future studies should assess the impact of motor/sensory loss on patients’ quality of life using appropriate scales. Clinicians should be aware of the potential for nerve injury and consider the associated injuries when making treatment decisions. Further research is necessary to clarify the mechanisms, identify predictors, and optimize treatments.

## Figures and Tables

**Table 1:** Baseline characteristics and results of studies on nerve injury in shoulder dislocation.

Study	Region	Type of Study	Follow-Up Period	*n*	*n* Nerve	Incidence	% Female	Diagnostic Criteria	Inclusion Criteria
Atef et al., 2015 [13]	Egypt	Prospective series	2011 to 2014	240	38	15.8	-	US +- MRI	Traumatic anterior glenohumeral dislocation
Gutkowska et al., 2018 [14]	Poland	Retrospective series	2000 to 2016	-	73	-	-	EMG +- MRI	Shoulder dislocation
Hardie et al., 2022 [5]	UK	Retrospective cohort	2016 to 2017	243	14	5.8	35.4	-	>18 yo shoulder dislocation
Jordan et al., 2021 [15]	UK	Retrospective series	2 years	-	28	-	-	Clinical	BPI and shoulder dislocation (>18 yo) who were managed within a specialist nerve injury unit over a period of 2 years
Kosiyatrakul et al., 2009 [7]	France	Retrospective series	2001 to 2007	-	14	-	-	EMG	BPI after shoulder dislocation
Pasila et al., 1978 [16]	Finland	Retrospective series	1973 to 1976	238	50	21.0	-	Clinical	Primary humeral dislocation
Perron et al., 2003 [17]	USA	Retrospective series	-	-	-	-	-	-	-
Robinson et al., 2012 [11]	UK	Prospective cohort	1995 to 2009	-	492	-	-	Clinical/ EMG for complex	Primary traumatic shoulder dislocation
Tiefenboeck et al., 2020 [18]	Austria	Retrospective series	2000 to 2016	15739	60	0.4	-	-	Shoulder dislocation and BPI or isolated nerve lesion and documented treatment details
Toolanen et al., 1993 [19]	Sweden	Prospective series	-	65	36/55	65.5%	44.6	EMG	-
Travlos et al., 1990 [20]	South Africa	Retrospective series	1980 to 1984	-	28	-	-	-	Patients were treated by the senior author in the brachial plexus
Visser et al., 1999 [6]	The Netherlands	Prospective series	31 months	93	37	39.8	41.9	EMG	Anterior dislocation of the glenohumeral joint
Yeap et al., 2004 [9]	Malaysia	Cross-sectional	1998 to 2000	100	11	11.0	26.0	Clinical	All anterior shoulder dislocations

BPI: brachial plexus injury.

**Table 2:** Assessment of the quality of studies through Methodological Index for Non-Randomized Studies (MINORS).

Study	Clearly Stated Aim	Consecutive Patients	Prospective Collection Data	Endpoints	Assessment Endpoint	Follow-Up Period	Loss Less Than 5%	Study Size	Adequate Control Group	Contemporary Group	Baseline Control	Statistical Analyses	MINORS
Atef et al., 2015 [13]	2	2	0	1	1	1	1	2	-	-	-	-	10
Gutkowska et al., 2018 [14]	2	2	0	1	1	2	1	2	-	-	-	-	11
Hardie et al., 2022 [5]	2	2	1	2	2	2	2	2	2	2	2	2	23
Jordan et al., 2021 [15]	2	1	0	2	2	2	1	2	-	-	-	-	12
Kosiyatrakul et al., 2009 [7]	2	2	0	2	2	2	1	1	-	-	-	-	12
Pasila et al., 1978 [16]	1	2	0	2	2	1	0	2	-	-	-	-	10
Perron et al., 2003 [17]	2	2	1	2	2	1	2	2	-	-	-	-	14
Robinson et al., 2012 [11]	2	2	2	2	2	2	1	2	2	2	2	2	23
Tiefenboeck et al., 2020 [18]	2	2	1	1	1	2	1	2	-	-	-	-	12
Toolanen et al., 1993 [19]	1	2	2	2	2	2	2	2	-	-	-	-	15
Travlos et al., 1990 [20]	1	2	0	1	1	2	1	1	-	-	-	-	9
Visser et al., 1999 [6]	2	2	2	2	2	2	0	2	-	-	-	-	14
Yeap et al., 2004 [9]	2	1	0	2	2	1	1	1	-	-	-	-	10

**Table 3:** Frequency and recovery rates of nerve injuries in shoulder dislocation: a meta-analysis of ten studies.

Injured Nerve	Number of Cases (Out of Total 826)	Frequency (%)	Associated Deficits
Axillary	289	35.0	Shoulder abduction and external rotation deficit
Radial	106	12.8	Wrist and finger extension deficit; elbow extension deficit; supination deficit
Suprascapular	92	11.1	Shoulder abduction and external rotation deficit
Musculocutaneous	85	10.3	Elbow flexion and supination deficit
Median	83	10.1	Wrist and finger flexion and opposition deficit; thumb opposition deficit
Global Plexus	78	9.4	Mixed deficits involving multiple nerves
Ulnar	77	9.3	Wrist and finger flexion and adduction deficit; thumb adduction and opposition deficit
Supraclavicular and Infraclavicular	8	1.0	Mixed deficits involving multiple nerves
Infraclavicular	7	0.9	Mixed deficits involving multiple nerves
Posterior and Medial Cord	1	0.1	Mixed deficits involving multiple nerves

**Table 4:** Frequency of greater tuberosity fracture and rotator cuff injury in patients with shoulder dislocation and nerve injury.

Study	Fracture of Greater Tuberosity (%)	Rotator Cuff Injury (%)
Atef et al., 2015 [13]	6.25%	6.25%
Gutkowska et al., 2018 [14]	23.3%	4.1%
Robinson et al., 2012 [11]	5.7%	2.1%
Travlos et al., 1990 [20]	7.1%	-

## Data Availability

The datasets analyzed during the current work are available upon reasonable request from the corresponding author.
